# “It’s just what’s around these days”: social and contextual pragmatism in substance transitions

**DOI:** 10.1186/s12954-025-01345-2

**Published:** 2025-11-26

**Authors:** Adams L. Sibley, David C. Colston, Elizabeth Joniak-Grant, Hillary L. Mortensen, Monica E. Swilley-Martinez, Brian W. Pence, Shabbar I. Ranapurwala

**Affiliations:** 1 Injury Prevention Research Center, University of North Carolina at Chapel Hill, 725 MLK Blvd, Chapel Hill, North Carolina 27599 USA; 2 Department of Health Behavior, University of North Carolina at Chapel Hill, 135 Dauer Dr, Chapel Hill, North Carolina 27599 USA; 3 Department of Epidemiology, University of North Carolina at Chapel Hill, 135 Dauer Dr, Chapel Hill, North Carolina 27599 USA

**Keywords:** Substance use, Polydrug, People who use drugs, Social cognitive theory, Qualitative

## Abstract

**Background:**

The overdose epidemic is presently driven by polydrug use, sparking renewed interest in why people initiate use of certain drugs or drug combinations. Current research privileges the physiological ends of consumption, often ignoring the social and environmental context of use. Framed by social cognitive theory, the purpose of this study was to characterize factors precipitating substance initiation, transition, and combination beyond the immediate effects of the substance(s).

**Methods:**

We conducted 30 semi-structured interviews with people who use drugs across North Carolina, exploring substance use history and risk and protective factors of polydrug use. Participants also completed a visual timeline activity. We used a staged analytic approach, beginning with deductive Structural Coding and ending with inductive Reflexive Thematic Analysis at both the transcript and excerpt levels.

**Results:**

We conceptualized substance transitions as pragmatic responses to environments of constraints and opportunities. Socially, transitions facilitated interpersonal closeness, aligned use with network norms, and responded to ubiquitous drug availability. Transitions also reflected navigation of material constraints, including which substances were locally available, logistically accessible, and financially sustainable.

**Conclusions:**

Beyond the desire for new or enhanced physiological effects, substance transitions serve social and practical functions, like facilitating emotional closeness and responding to market conditions. Interventions to reduce the risks of use should expand viable options by addressing structural barriers and promoting safe, affordable, and accessible supply.

## Introduction

Between 1999 and 2022, more than one million people died from overdose in the United States, with annual mortality rates growing 6.4-fold in this time [1, 2]. The overdose epidemic has evolved from a crisis of prescription opioid misuse to periods of increasing use of heroin followed by illicitly manufactured fentanyl [3]. The United States has now entered a fourth wave of the overdose epidemic defined by polydrug use (the use of more than one substance in a short period of time) [4]. In 2017–2019, 81% of people who used opioids also used other drugs in the previous month [5]. Since 2020, cocaine, methamphetamine, and benzodiazepines—frequently used in combination with fentanyl—have surpassed heroin in drug-involved mortality [1].

Given the acute risks of polydrug use to health, especially increased risk of overdose [6, 7], researchers have had a renewed interest in examining why people use certain drugs or combinations of drugs [8]. Several qualitative studies have documented that the choice of substance(s) is primarily driven by desired or expected effects; in the case of polydrug use, for instance, substances may be combined to enhance or prolong a high (e.g., combining opioids and benzodiazepines), to counteract effects (e.g., taking stimulants to wake up after opioid consumption), or to ease symptoms (e.g., using prescription opioids to stave off withdrawals from heroin) [5, 9, 10]. Likewise, research on motives for using single drugs has focused on their physiological effects, e.g., to relieve pain, achieve a pleasurable high, increase energy, or regulate difficult emotions [11–14], often driven by personal circumstances like depression, anxiety, trauma, or social disadvantage [15–17].

These perspectives suggest that substance selection is a process of matching psychological or psychosocial ends with physiological effect, such as using substances to manage physical pain, enhance mood, or cope with difficult emotions. This line of reasoning captures two of three determinants defined by Norman Zinberg’s “drug, set, and setting” framework: the pharmacology of the substance itself (‘drug’) and the personal attributes of the user (‘set’) [18]. Substantial scholarship has examined how the third determinant—physical and social ‘setting’—shapes drug use practices, most notably through Rhodes’ risk environment framework demonstrating how physical, social, economic, and policy environments interact to produce drug-related harms [19, 20], and Duff’s theorization of contexts as active assemblages of space, embodiment, and practice [21]. Recent literature has further demonstrated how structural vulnerabilities and socio-economic inequities shape polydrug use patterns, particularly opioid-stimulant combinations, as individuals navigate poverty, housing instability, incarceration, overpolicing, and unmet healthcare needs [22–25]. While these frameworks have been invaluable in revealing how environments structure risk and shape the textures of drug use, they tend to view context primarily as a pathway to harm. Less explored is how environments also structure situated possibilities, within which individuals act as pragmatic actors. From this perspective, substance selection is not inevitably oriented toward harm but reflects responsive choices aligned with people’s circumstances and priorities. We use the term pragmatism to capture these situated, reasoned responses to environmental conditions—whether shaped by social relations, material realities, or other contextual factors. By foregrounding pragmatism, this study extends the “setting” dimension of Zinberg’s framework to highlight how environments both constrain and enable behavior. Building on this foundation, and informed by social cognitive theory’s emphasis on reciprocal determinism between person, behavior, and environment [18, 26], this study examines factors precipitating substance transitions beyond immediate physiological benefits.

According to Bandura’s social cognitive theory (SCT), behavioral patterns like drug use trajectories are reciprocally determined by personal factors (i.e., genetics, cognitions, affect) and environmental factors (i.e., social norms, physical setting, environmental cues) [26, 27]. While risk environment frameworks also acknowledge reciprocal relations between individuals and environments [19], SCT distinctively emphasizes how people navigate their circumstances through both conscious and habitual responses. The theory recognizes that behaviors emerge from a mix of intentionality, learned patterns, social modeling, and immediate responses to environmental cues [28]. This focus on pragmatic navigation distinguishes SCT from frameworks that primarily trace pathways from structural conditions to harm.

In this qualitative study, we look beyond physiology to explore how individuals navigate substance use trajectories within their social and material contexts, including initiation, reinitiation, transitions between substances, and adoption of new substances within polydrug use.

## Methods

### Sampling and recruitment

This research was part of a larger study to explore the risk and protective factors related to polydrug overdose in North Carolina. The qualitative data set included interviews with people who use drugs, harm reduction professionals, and treatment providers; only data from people who use drugs were used in the current analysis. Eligibility criteria for participants (*n* = 30) included past 30-day use of any illicit opioid or use of any prescription opioid not as prescribed; current residence in North Carolina; and age of 18 years or older.

We used purposive sampling to ensure a range of perspectives and experiences relevant to understanding substance use transitions. We sought diversity across several characteristics: current polysubstance use (i.e., meeting eligibility and also using at least one other opioid or non-opioid drug, excluding tobacco, in the past 30 days); gender; race/ethnicity; age (including those 50 years or older); and prescription opioid history. This sampling strategy was designed to capture varied experiences with substance transitions rather than to enable subgroup comparisons. Recognizing that drug markets and demographics vary regionally, participants were recruited from all three regions of North Carolina (Mountains, Piedmont, Coastal Plain).

Syringe service programs served as the primary recruitment sites; study staff contacted SSP leadership through established networks or a public contact database for permission to recruit on-site. Recruitment was also conducted in areas known to be frequented by PWUD (e.g., public parks) and by participant referral.

### Data collection

Co-author DCC conducted semi-structured in-depth interviews with participants between July 2023 and November 2023. Interviews took place in harm reduction offices or a location of participants’ choosing, including quiet spaces in fast food restaurants and public parks. Participants first completed the consent process and a brief demographic questionnaire. The interview then opened with an icebreaker question and a visual timeline activity. Visual timelines can help elicit autobiographical memories, organize thoughts, and structure sequences of events [29, 30]. Participants were asked to “think about your journey—the important events or periods in your life that shaped who you are today” and draw a timeline. Generic sample timelines, markers, and blank paper were provided. Participants were given eight minutes and told that no particular structure nor content was expected. They were encouraged to refer or add to the timeline throughout the interview.

The remainder of the interview guide covered the following topics: past and present drug use (e.g., drug(s) used, context of first use, feelings about use), drug transitions (e.g., context of transition, feelings about transition), polydrug use (e.g., attitudes and experiences, reasons for use or non-use, perceived local trends), and risk perceptions (e.g., overdose, infectious disease and other harms, drug adulteration). Interviews lasted 66 min on average (range: 25–126), and participants were reimbursed $30 for their time. All procedures received ethical approval from the UNC-Chapel Hill Institutional Review Board (#23–0808).

### Data analysis

Interviews were audio-recorded, transcribed verbatim by a transcription service, and uploaded to a password-protected institutional server. Transcripts and demographic data were imported into Dedoose v9.0.

We used a staged approach to data analysis. First, the coding team (ALS, DCC, EJG, HLM, MESM) developed and applied a structural coding framework [31] to organize and index major topical areas across transcripts. This initial codebook was developed iteratively over three team meetings, with topics and definitions developed by consensus. Codes included ‘intentional polysubstance use,’ ‘unintentional polysubstance use,’ ‘drug use transitions,’ ‘overdose experiences,’ ‘protective strategies,’ ‘social context of use,’ and others. The team double-coded two transcripts to check for consensus and resolve discrepancies, then ALS, DCC, and HLM single-coded and memoed on the remaining transcripts.

The team met regularly throughout this initial coding phase to discuss emerging themes. One particularly salient pattern was that participants described multiple factors influencing their transitions between substances beyond the pharmacological properties of the drugs themselves. Based on this observation, we developed the following research question for deeper analysis:

What might be factors precipitating transitions between substances, including transitions into polydrug use, beyond the immediate physiological benefits of these substances?

We then conducted a second round of analysis using Reflexive Thematic Analysis [32] to explore this specific question. Through close reading of transition narratives, we recognized that participants’ accounts reflected the inseparable nature of person, behavior, and environment—transitions were simultaneously individually determined and environmentally shaped. This observation led us to adopt social cognitive theory as an interpretive lens, particularly its principle of reciprocal determinism whereby behavior emerges from the dynamic interplay between personal agency and environmental constraints [28]. This lens allowed us to recognize participants’ accounts not as stories of pure choice or pure constraint, but as pragmatic navigation within structured possibilities. Throughout our analysis, we applied SCT’s triadic framework of person-behavior-environment to understand how substance transitions emerged at these intersections.

Our subsequent inductive coding captured this bidirectional relationship: codes such as ‘substance choices shaped by social bonds,’ ‘adapting to market changes,’ and ‘ease of access as priority’ reflected pragmatic choices made within, rather than despite, environmental and interpersonal contexts.

Two coders (DCC and ALS) applied this codebook to two transcripts, then met and discussed coding decisions until consensus was reached on definitions and code applications. ALS then coded the remaining transcripts. We next exported code reports from Dedoose into Microsoft Excel and discussed emergent themes. Finally, we identified and named our themes, organizing relevant excerpts within these themes in Excel to prepare the data for presentation.

In the results below, pseudonyms and pseudonymous ages are presented throughout.

## Reflexivity

The analysis team maintained reflexivity through regular discussions about our varying interpretations and assumptions during team meetings. The primary analyst (ALS), while bringing extensive qualitative research experience and professional expertise in harm reduction, has limited personal experience with the substances discussed and thus cannot speak firsthand to the interplay of agency and environment in substance use decisions. This outsider perspective was both a limitation and an asset—allowing analytical distance while requiring careful attention to participants’ own descriptions of their experiences.

The third author (EJG) played a crucial role in challenging initial interpretations that framed substance use decisions as discrete, rational choices. Through team discussions, we recognized that participants’ narratives revealed decision-making as messy, constrained, and often occurring under duress—leading us to adopt a more interpretivist approach that honored the complexity and ambiguity in participants’ accounts. This shift was essential in developing our understanding of ‘pragmatic’ choices as those made within, rather than despite, environmental and social constraints.

## Results

Thirty participants were interviewed, with a median age of 38 (range: 20–71). Participants identified as non-Hispanic White (*n =* 20), African-American/Black (*n =* 8), or mixed race (*n =* 1), with one participant not reporting racial/ethnic identity. Slightly more than half (*n =* 17, 56.7%) were men and the remainder were women (i.e., no additional genders were reported). Participants resided in 10 counties across the three regions of North Carolina: Mountains (*n =* 1), Piedmont (*n =* 18), and Coastal Plains (*n =* 11).

Below, we describe contextual factors that precipitated substance transitions—specific moments when participants initiated new substances or reintroduced substances to their repertoire. We conceptualize these processes as *pragmatic* as they are conditioned on environments of constraints and opportunities. Participants navigated their social and material contexts strategically, making transitions that reflected both environmental limitations and practical priorities. We organize these factors by the environmental conditions that shaped transitions, recognizing that the same contextual factors may precipitate different types of transitions for different individuals.

## Social pragmatism

Participants’ substance transitions often reflected pragmatic responses to their social environments. These transitions represented practical adaptations to social realities: maintaining relationships, navigating group dynamics, or managing life within drug-saturated social networks. Some participants transitioned to new substances as a means of bonding with friends or partners; others adopted substances prevalent in their social circles to maintain belonging; and many navigated environments where drug use was so ubiquitous that transitions became a pragmatic response to constant exposure. Below we describe three social forms of pragmatism in substance transitions: interpersonal bonding, fitting in, and accepting the social ubiquity of use.

### Interpersonal bonding

Participants explained that transitioning to new drugs established or reinforced emotional closeness with friends, family, or significant others. This pattern was common in adolescence, when most participants used their first drug with peers, an older sibling, or a parental figure, but persisted into adulthood. Some participants described tight-knit friend circles that used the same drugs together (“It was always us four…just through thick and thin,” 30 M), and the transition into new drugs often reflected a desire for belonging:

INTERVIEWER: How did you start using, uh, crack from the powdered cocaine?

PARTICIPANT: Just the people I’m around…I just wanted to try it because…You always used together. I’d go and see my friend. And we just was bonding. *59 F*.

In many cases, drug transitions occurred in intimate relationships with partners who also used drugs. Often one partner would relapse (e.g., after running into an old dealer), and the other would follow suit. These participants described feeling their shared drug use reflected codependence (i.e., maladaptive bonding). In other cases, transitions presented a welcome opportunity for closeness. Michael, 28, had been using heroin since he was kicked out of his methadone program. He only ever used meth “here and there … depending on who I was around,” until he met Shelly, who became like an aunt to him. Shelly also happened to sell meth, which she would offer to Michael for free. Eventually it became his second drug of choice, a product of both economic and interpersonal incentive:

I got it for free, and just kinda got stuck in that little clique or, um, whatever you wanna call it. I mean, you know, she was feeding me drugs but also was, you know, one of the only people that was there for me when I got out of prison the first time. *28 M*.

Michael’s transition to regular meth use illustrated how practical considerations—free access, social support—intersected to shape substance trajectories. Though Shelly has since moved out of town, Michael notes that “I could call her and she’d be there for me,” highlighting how the relationship outlasted the substance transition it facilitated.

### Fitting in

Substance transitions often occurred within social environments where particular drugs were normative. Participants described adapting their substance use to align with established practices in their social circles—a pragmatic response to maintain belonging and navigate group expectations: “Being around different people and, uh, you wanting to fit in, so you kinda like, follow the lead—You do what they’re doing,” *57M.* Several participants explicitly identified their substance choices as responses to what was normative in their immediate environment (e.g., “If people around me weren’t doing Percs [Percocet], I probably wouldn’t have started doing Percs,” *33M*). Others felt a pressure to use the popular drugs in their social circles, including a desire to use multiple substances because “they don’t wanna be left out, they don’t wanna feel alone” (*50M*).

Renee’s trajectory illustrates how transitions can serve purely social functions disconnected from individual circumstances. Though she had experienced significant trauma throughout her life, when asked what precipitated the addition of heroin to her existing cocaine use, she was clear: “I just wanted to try it. Nothing going on. I didn’t have no hard time. Nothing like that… I just did it—what you call it—to fit in” (56 F). Her friends were already using heroin, and when she discovered her boyfriend was as well, the transition felt inevitable: “So that’s what made me try that, because my friends was already doing it. So once I realized he was doing it, I tried it.” Years later, Renee uses fentanyl and cocaine purely due to dependence. She lamented that she no longer has friends—her substance use had outlasted the social context that initially precipitated it.

Wayne, 71, never touched a drug outside alcohol until he was 42, when he started freebasing cocaine. Recently, he’s begun to use fentanyl, a change that he attributes to norming among his friends:

It goes back to who you’re hanging around with, you know, who you’re associating with…As long as you’re associated and…it’s right there in front of you and being offered to you and, at that moment, your life is maybe topsy-turvy, so why not? But that ‘why not’ brings on a whole bunch of other shit. That ‘why not’ can be the thing that sends you to another part of the world. But yet, still, at that moment in time, that’s your best solution because of peer pressure or whatever. *71 M*.

Drug transitions could happen in licit contexts as well. Jake, 31, already used unregulated opioids and “nerve pills” when he took a job working construction, a setting where drinking on the job is normative. After joining this workplace ritual, alcohol became part of his routine:

So I went to drinking and started drinking beer. I drank beer heavily for a while. And then I went to cheap vodka, Aristocrat Vodka, which I still enjoy to this day…And, you know, alcohol’s one of them things that can get outta hand real quick. *31 M*.

Jake’s transition illustrates how environmental norms in one context (workplace) can precipitate substance adoption that extends beyond that setting—once introduced through work culture, alcohol escalated and became integrated into his broader polydrug pattern.

### Social ubiquity

For some participants, substance transitions occurred within environments where drug use was pervasive. Rather than actively seeking new substances, participants described responding to consistent exposure through routine social interactions. In these drug-saturated networks, substances were offered by acquaintances, dealers made themselves known, and opportunities presented themselves without pursuit: “No matter where, they’re gonna be everywhere…Eventually, you’re gonna run into somebody. That’s how we meet people down here. They come up to you. You don’t gotta go look for them. They come to you.” *(32 F)*.

Social ubiquity led some participants to engage in polydrug use or transition into riskier substances, as was the case for Sam, 40. Though he attributes the transition from alcohol to opioids to his divorce and custody battle, he admits that the transition was only possible because of social opportunity:

Eventually you hang out with drug users long enough…you’re gonna get close enough to where they’re gonna be like, “Hey. Do you want a hit? Do you want a bump?“…It’s not like I went to someone on the street, like, you know, “Hey man. Do you have some fentanyl?” …So it just kind of happens. It’s not something that you make happen or seek. *40 M*.

One participant described the transition into polydrug use as a “natural progression” within a social milieu where multiple substances circulated. Rather than constrain choice, ubiquity structured the field of possibilities, which the participant likened to the options on a coffee shop menu:

You know, if you do one drug, you’re in a group of people that use all sorts of other drugs, you’re gonna try them eventually…Nobody’s hanging out with a group of people that only do cocaine, you know, and only have access to cocaine. *38 F*.

## Contextual pragmatism

For many participants, substance transitions reflected pragmatic navigation of material constraints. Participants weighed competing factors—which substances existed in local markets, how easily they could be obtained, and what they could afford—making transitions that aligned with their circumstances and priorities. Almost all participants engaged in *contextual pragmatism*, where consumption patterns emerged from strategic responses to environmental resources and limitations.

### Availability

Participants’ substance choices reflected what circulated in local drug markets. Many described adapting to shifting supply, using particular substances because “it’s just what’s around nowadays” (*34M*). Some participants described moving from a controlled environment (e.g., treatment program) back into the community and needing to find a new substance to stave off withdrawals or maintain desired psychoactive effects. This was the case for a participant whose first encounter with opioids was using buprenorphine nonmedically in prison and who transitioned to heroin on the outside to cope with life stressors. However, the most common examples of this theme reflected shifting drug markets. Some participants described transitioning from opioids to methamphetamines because the latter became more widely available (e.g., due to supply issues during COVID or dealers’ reluctance to sell opioids due to the state’s Death by Distribution law, which allows a person who provided drugs to someone who died to be charged with second-degree murder). Market transitions could even be seasonal:

Pills, it started with pills. I was using Somas [muscle relaxant] and Klonopins [benzodiazepine] and Seroquel [atypical antipsychotic]. Then it went to heroin. Then it went to pills again, and then it went to methamphetamine, and I went on that. It’s a tight work. It was the seasons. Like, we have four seasons here. Drug sales have seasons too, you know? *46 M*.

This participant’s cyclical pattern illustrates how some individuals tracked and adapted to predictable market fluctuations.

More commonly, participants experienced a trajectory of use shaped by national trends in illicit opioid markets: from pills to heroin and from heroin to fentanyl. These transitions might include decisional factors like price and potency, or adapting to market realities, shifting to whatever substances were available (“Whatever you can get your hands on at the moment…you’re gonna do it,” *20M*).

Jimmy described how COVID-era market shifts shaped his substance transitions. His drug of choice was always opioids, starting with black tar heroin before switching to fentanyl. Though he had dabbled with meth in the past, he never used it regularly. However, when meth became more widely available in his area during the pandemic, he adapted his use accordingly:

“That’s actually when I started using ice [crystal methamphetamine] really, really, really heavy…I got more plugs, and I was getting it cheaper…[My area] was being flooded with ice. You know, a lot of times you had to go to [a nearby city] to get it or, you know, [another nearby city]…it became easier to get in the area because the only transportation I had for a short period of time was a scooter.” *41 M*.

This participant’s account illustrates how individuals tracked local market conditions and adapted their substance use based on both supply increases and practical transportation constraints.

While many participants adapted reactively to market shifts, others were more proactive about choosing substances based on availability. This may involve having a ‘backup’ at the ready in case their drug of choice is unavailable (e.g., meth for cocaine). For such participants, aligning their substance choice with what circulated in their social networks ensured reliable access when needed:

Just everyone around me, this is what they use…You know, you don’t wanna really get something, like, off the wall and, when you need something, no one else around you has it. You know what I mean? So you kinda, like, wanna stick with what majority uses. You know, ‘cause, if you need help, you can always, you know, go to friends and get some help. *50 M*.

### Accessibility

Beyond whether substances existed in local markets, transitions reflected calculations about logistical convenience. Multiple participants described transitioning from prescribed to illicitly obtained substances when navigating the prescription system became more burdensome than street access. One participant explained:

Just having to go to the doctor all the time and try to get, you know, a prescription and stuff was, like, a hassle when I could see somebody on the corner and get what I needed. You know I mean? It was just out of convenience. *49 M*.

Another participant’s account illustrated how multiple accessibility barriers—transportation, pharmacy stock, distance—could compound to make street access more practical:

So [my significant other] just stopped going to get her [Subutex] prescription every month. We just started buying heroin ’cause it’s cheaper, really, and easier. Not really easier. I mean it kinda was though in a sense, um, because we had to go all the way to…way in town somewhere to get it. Then we had to go all the way to a pharmacy. Sometimes they don’t have that. They don’t have these. They don’t have enough…It’s a lot, especially if you ain’t got a car. If you’re riding buses around there, it’s hell. *27 M*.

Two unrelated participants who used opioid pills while selling heroin recognized the inefficiency of maintaining separate sources and made the same pragmatic shift:

I was popping pills, and I was selling dope, so I went like, “Ain’t no point in me keep-keep, um, buying these pills when I got pretty much the same thing in my pocket.” *44 M*.

For these participants, accessibility shaped not only temporary substitutions but lasting changes in substance use patterns.

### Affordability

Finally, participants made transitions that aligned with their financial resources, with cost considerations shaping which substances they could sustain over time. As with availability, the most common transitions were from opioid pills to heroin and from heroin to fentanyl:

The amount of money that I was spending, you know? There’s nothing for me to eat, you know, 20, 30, 40 Lortab 10s a day or, you know, Percocet 10s a day…Just got super duper expensive, and somebody said, hey, you know, John [pseudonym], you can get that shit a hell of a lot cheaper, you know, or save yourself a hell of a lot of money and just [use heroin]. *42 M*.

I found myself spending more and more money. And then, that’s when we found out about fentanyl, that you can spend less money, and you can get a better high, I guess, and it suppresses your emotional side better, and you’re not spending as much money. *31 F*.

Others transitioned between substance classes. Darrell, 44, started using ecstasy in his early 20s to curb his inebriation from alcohol. After adding cocaine to his repertoire, he entered a methadone program as a cost-saving measure: “cheaper—$15 a day as opposed to whatever X amount I was spending at the time,” *44M*; he would later transition to heroin and fentanyl.

Some participants adopted polydrug combinations as strategies to manage limited resources. Carla, 43, developed a polydrug strategy to stretch limited fentanyl supply by combining it with alcohol to enhance effects:

I didn’t have enough dope. I could drink and it would make it stronger. You know? Um, so that’s probably why my alcohol use was heavy right in here, too, um, because, like, if I only had the availability of, like, a lesser amount, then I would usually use. If I drank before I use it [fentanyl], it would have more of an effect. *43 F*.

Carla’s account illustrates how financial constraints shaped not only which substances participants used, but how they combined substances to maximize limited resources.

The various dimensions of contextual pragmatism often intersected in complex ways. One participant described how prioritizing affordability created new logistical challenges that precipitated polydrug use:

PARTICIPANT: We used to just do, like, fent. We didn’t do coke with it. But the reason why we started doing the coke was because we were having to drive from here to [another city] every, like, three days to rig up. And we would drive four hours later. Sometimes we had to wait on our dealer for, like, two or three hours. And then we’d grab it and then we’d drive four hours back. So it was, like, a long fuckin’ day of driving. And to keep us up we would do the coke from there back, and it just kinda became a habit of doing it with it. So, yeah. That’s kinda how that started.

INTERVIEWER: Um, if you don’t mind my asking, uh, is there a particular reason you go to [another city] for it?

PARTICIPANT: Prices are way better. *30 M*.

This account illustrates how participants navigated multiple material constraints simultaneously, with pragmatic responses to one constraint (driving for cheaper prices) generating new challenges (fatigue from a long journey) that required additional adaptation (adding cocaine, which was available locally). In such cases, substance transitions reflected participants’ negotiation of interconnected practical realities.

## Discussion

In this qualitative study, we explored factors precipitating substance transitions beyond the immediate physiological benefits of use. Participants described pragmatic responses to their social and material environments. Socially, transitions facilitated interpersonal closeness, aligned use with microsocial norms, and responded to the ubiquity of drugs within social networks. Transitions also reflected navigation of material constraints including which substances circulated in local markets, logistical barriers to access, and financial considerations. Participants rarely emphasized the pharmacological effects of substances when describing transitions; rather, they described strategic responses to environmental conditions. Some transitions occurred within relationships offering emotional support, as with the participant who adopted alcohol use to bond with construction coworkers. Others reflected navigation of volatile drug markets or transportation barriers that made street access more feasible than pharmacy access. These patterns have been corroborated by studies on emergent substances in China [33] and recent methamphetamine trends in the United States [34].

As SCT’s principle of reciprocal determinism suggests, people are as much shapers as products of their environment, able to mold, circumvent, and adapt to contextual constraints [28]. This was evident among participants who navigated structural barriers like pharmacy inaccessibility and transportation limitations, making adaptations like switching from prescribed medications to street drugs when the latter proved more feasible. Others retained social ties that aligned with their priorities for use, or attempted to reshape their environments entirely by relocating or severing triggering social ties.

Our findings extend risk environment frameworks by foregrounding pragmatic navigation rather than structural determination. While risk environment scholarship has been invaluable in demonstrating how physical, social, economic, and policy contexts structure harm, these frameworks can inadvertently position people who use drugs as passive subjects of environmental forces. Our analysis centers a more complex reality: participants actively navigated constrained environments, making strategic assessments about social relationships, market conditions, and logistical barriers. This perspective honors both the reality of structural marginalization and the agency people exercise within it. Substance transitions reflect neither unconstrained choice nor environmental determinism, but rather responses to situated possibilities—what people can feasibly do given their circumstances and priorities. Though risk environment scholarship acknowledges pragmatism and agency [19], it has tended to foreground how environments produce harm; our analysis emphasizes how people navigate these constraints, offering a complementary lens on substance transitions. This reframing has important implications: if transitions emerge from strategic navigation of constraints, interventions should focus on expanding the range of viable options with existing constraints (e.g., improving pharmacy access, ensuring predictable supply) while simultaneously working to dismantle structural barriers (e.g., criminalization, healthcare inequities) that create these constraints in the first place.

We recognize important limitations in applying a pragmatism lens. Not all transitions described by participants reflected strategic navigation—some occurred under coercion or severe constraint that left little room for pragmatic choice. For instance, one participant initiated heroin use within an abusive relationship characterized by isolation and emotional manipulation; another described accessing only one available substance while incarcerated. In such cases, framing transitions as ‘pragmatic’ risks obscuring the violence and structural constraints that fundamentally limit agency. These examples remind us that risk environment frameworks remain essential for understanding how power inequities, interpersonal violence, carceral systems, and material deprivation determine many aspects of substance trajectories. Our analysis does not dismiss these realities or suggest that people always exercise meaningful agency within constrained environments. Rather, we argue that pragmatism and constraint exist on a continuum: some transitions reflect strategic navigation of limited options, while others occur under conditions so constrained that agency is minimal or absent. By illuminating the pragmatic dimensions of substance transitions, we aim to complement—not replace—analyses of structural oppression and interpersonal violence. Both perspectives are necessary: acknowledging how people navigate constraints when possible, while recognizing when constraints become so severe that navigation itself is foreclosed.

Existing research on substance selection typically emphasizes the functional benefits of the substance itself within the triad of behavior, self, and environment [8]. For instance, Boileau-Falardeau et al.’s rapid qualitative review of polysubstance use identified only physiological motivators like enhancing a high or counteracting effects in included studies, though the authors concede that use is not always a matter of choice and discuss the influence of social and legal environments [8]. While motivations for whether to use drugs are often understood as environmentally shaped (e.g., coping with trauma, responding to social disadvantage), the choice of which substance to use has been framed primarily as pharmacologically driven. Our findings challenge this distinction, demonstrating that substance selection is also shaped by social and material environments. Participants described transitions that served social functions (facilitating relationships, aligning with group norms) and reflected material constraints (market availability, transportation barriers, cost) as often as they mentioned drug effects. Our findings reveal that substance transitions are neither purely matters of individual choice nor environmental determination, but emerge at their intersection. This perspective moves beyond contentious debate about agency versus constraint [35] and extends motivational models of substance use by examining not just psychological drivers (e.g., using to fit in or cope) but how social and material environments structure which responses become pragmatically viable [36–38].

One key distinction merits attention: factors precipitating substance transitions may differ from those sustaining continued use [39, 40]. But the implications for policy and practice are different for each. Understanding why people initiate or adopt new substances suggests opportunities to address structural barriers before use patterns become entrenched. Rather than intervening solely at the interface of self and behavior (e.g., educating about physiological harms), interventions can expand viable options at the interface of self and environment—addressing the accessibility, affordability, and social network conditions that make certain transitions reasonable given people’s circumstances.

Our findings suggest potential structural interventions to expand viable options. For instance, participants who transitioned from legally prescribed drugs to unregulated opioids described multiple accessibility factors: transportation to clinics, pharmaceutical stock shortages, and bureaucratic requirements. These transitions reflected responses to structural constraints rather than solely preference for unregulated opioids like fentanyl. Addressing such barriers could reduce transitions driven by logistics rather than pharmacology. For instance, mail-order buprenorphine—legal under the Controlled Substances Act, though regulated variably by state [41] – remains underutilized, with fewer than 16% of adult prescription users accessing mail-order pharmacies in 2018 [42]. This untapped resource may be suitable for people who face barriers to accessing local brick-and-mortar pharmacies, including insufficient stock and pharmacist stigma [43, 44]. However, research is needed to better understand current mail-order MOUD dispensing rates and barriers to expanding access to these services such as a lack of permanent address.

Interventions are also needed to address how social environments structure drug transitions (“Of all the stimuli in our environment, it is other people that arguably have the largest impact on our behavior” [45]). Many participants described transitions shaped by network composition, relationship dynamics, and the ubiquity of polydrug use within their social milieus. Structural approaches like safe supply [46] might ensure reliable access to preferred substances, reducing dependence on social networks where polydrug use is normative and unpredictable supply creates pressure to accept whatever is available. Individual-level harm reduction strategies could address transitions that occur in social settings—for instance, having one’s drug of choice readily available provides a default option in social contexts where polydrug use is common, reducing reliance on whatever substances others offer. Interventions could also target social networks. Previous studies combined motivational interviewing with social network visualization to help people in recovery to identify individuals who support or undermine recovery goals [47, 48]; in harm reduction contexts, such tools could help individuals in identifying social contacts most likely to encourage experimentation and new drug use, as well as contacts with whom use is more likely to be stable and predictable. Further research using dynamic network analysis [49] and agent-based modeling [50] could illuminate how substance transitions propagate through social networks, informing network-level interventions.

### Limitations

Participants were mainly recruited through harm reduction organizations; our findings may not fully capture the experiences of people who use drugs who do not engage in health services and may have different reasons for substance transitions. Our sample had notable gaps in representation. No American Indian participants were recruited despite North Carolina’s significant American Indian population, who may experience unique substance use contexts related to historical trauma and rural residence. The Mountain region was represented by only one participant, limiting our ability to capture the drug market dynamics of Appalachian communities. These populations may experience different pathways to substance transitions that our study could not adequately explore. These findings also do not inform solutions for unintentional polydrug through the consumption of adulterated substances, a phenomenon that poses grave health risks to people who use drugs. Finally, our findings do not examine the macro-environment, especially the sociolegal context of drug criminalization. The choice of substances is intrinsically shaped by *de jure* constraints on possession and consumption, restricted access to controlled medications, and criminal penalties that often push people into riskier use [51–53].

Our analysis focused on factors precipitating transitions rather than the protective dynamics within drug-using communities. We acknowledge that our framing does not capture the harm reduction culture of care that exists within these networks—the mutual aid, resource sharing, and protective practices through which people who use drugs support one another’s safety and wellbeing [54, 55]. Research should continue to examine both how social networks facilitate transitions and how they function as sites of collective care and harm reduction practice, moving beyond deficit-focused analyses to recognize the adaptive and prosocial aspects of drug-using communities.

## Conclusion

This qualitative study explored factors precipitating substance transitions beyond immediate physiological benefits. Many such transitions reflected strategic navigation of social and material constraints, including network dynamics, market conditions, logistical barriers, and financial limitations. Our findings extend risk environment frameworks by emphasizing that transitions reflect neither individual choice nor environmental determinism, but rather pragmatic responses to situated possibilities—what people can feasibly do given their circumstances. In an era of unpredictable drug markets and polysubstance use [4, 56], understanding how people navigate these environments can inform structural interventions that account for the constraints shaping substance use patterns.

## Data Availability

Data may be made available upon reasonable request to the corresponding author.
